# One-Pot Synthesis of Amphiphilic Biopolymers from Oxidized Alginate and Self-Assembly as a Carrier for Sustained Release of Hydrophobic Drugs

**DOI:** 10.3390/polym14040694

**Published:** 2022-02-11

**Authors:** Zhaowen Liu, Xiuqiong Chen, Zhiqin Huang, Hongcai Wang, Shirui Cao, Chunyang Liu, Huiqiong Yan, Qiang Lin

**Affiliations:** 1 Key Laboratory of Tropical Medicinal Resource Chemistry of Ministry of Education, College of Chemistry and Chemical Engineering, Hainan Normal University, Haikou 571158, China; liuzhaowenyifan@126.com (Z.L.); chenxiuqiongedu@163.com (X.C.); whcz0505@163.com (H.W.); 2 College of Pharmacy, Gannan Medical University, Ganzhou 341000, China; hzqzll@126.com; 3 Key Laboratory of Natural Polymer Functional Material of Haikou City, College of Chemistry and Chemical Engineering, Hainan Normal University, Haikou 571158, China; caoshirui@163.com (S.C.); liuchunyang212021@126.com (C.L.); 4 Key Laboratory of Water Pollution Treatment & Resource Reuse of Hainan Province, College of Chemistry and Chemical Engineering, Hainan Normal University, Haikou 571158, China

**Keywords:** amphiphilic conjugates, sodium alginate, nanocarrier, self-assembly, sustained release

## Abstract

In this paper, we developed an organic solvent-free, eco-friendly, simple and efficient one-pot approach for the preparation of amphiphilic conjugates (Ugi-OSAOcT) by grafting octylamine (OCA) to oxidized sodium alginate (OSA). The optimum reaction parameters that were obtained based on the degree of substitution (DS) of Ugi-OSAOcT were a reaction time of 12 h, a reaction temperature of 25 °C and a molar ratio of 1:2.4:3:3.3 (OSA:OCA:HAc:TOSMIC), respectively. The chemical structure and composition were characterized by Fourier transform infrared spectroscopy (FTIR), ^1^H nuclear magnetic resonance (^1^H NMR), X-ray diffraction (XRD), thermogravimetry analyser (TGA), gel permeation chromatography (GPC) and elemental analysis (EA). It was found that the Ugi-OSAOcT conjugates with a CMC value in the range of 0.30–0.085 mg/mL could self-assemble into stable and spherical micelles with a particle size of 135.7 ± 2.4–196.5 ± 3.8 nm and negative surface potentials of −32.8 ± 0.4–−38.2 ± 0.8 mV. Furthermore, ibuprofen (IBU), which served as a model poorly water-soluble drug, was successfully incorporated into the Ugi-OSAOcT micelles by dialysis method. The drug loading capacity (%DL) and encapsulation efficiency (%EE) of the IBU-loaded Ugi-OSAOcT micelles (IBU/Ugi-OSAOcT = 3:10) reached as much as 10.9 ± 0.4–14.6 ± 0.3% and 40.8 ± 1.6–57.2 ± 1.3%, respectively. The in vitro release study demonstrated that the IBU-loaded micelles had a sustained and pH-responsive drug release behavior. In addition, the DS of the hydrophobic segment on an OSA backbone was demonstrated to have an important effect on IBU loading and drug release behavior. Finally, the in vitro cytotoxicity assay demonstrated that the Ugi-OSAOcT conjugates exhibited no significant cytotoxicity against RAW 264.7 cells up to 1000 µg/mL. Therefore, the amphiphilic Ugi-OSAOcT conjugates synthesized by the green method exhibited great potential to load hydrophobic drugs, acting as a promising nanocarrier capable of responding to pH for sustained release of hydrophobic drugs.

## 1. Introduction

Polymer nanocarriers made from naturally occurring and biodegradable polymers have attracted much attention, especially in various drug delivery systems, since they offer a promising means by which to enhance the therapeutic values of drugs by improving their bioavailability, solubility and retention time, as well as benefitting patients due to lower cost and reduced toxicity [1,2,3,4,5]. Through great efforts from researchers, various polymer nanocarriers are currently being developed with the aim of improving drug delivery, especially of hydrophobic drugs including liposome, polymeric nanoparticles, self-assembly micelles, polymersomes, polyelectrolyte complexes, polymer–drug conjugates, dendrimers and others [6,7,8,9]. Among them, polymeric micelles with inbuilt unique features to solubilize insoluble drugs present one of the promising drug delivery candidates and have been proven to have a multitude of advantages, such as simple, convenient and time-saving synthetic procedures, prolonged circulation of the blood and biocompatibility, low cytotoxicity, efficient drug delivery and controlled release [10,11,12].

Polymeric micelles are formed by amphiphilic block copolymers with hydrophilic and hydrophobic units, which can self-assemble in aqueous media above the critical micelle concentration (CMC) into unique and stable core-shell structures and are driven by a decrease in interfacial free energy [13,14]. Their hydrophobic core promotes the solubilization of water-insoluble drugs, protecting them from degradation by harsh environments, whereas their outer hydrophilic shell can reduce the binding of plasma proteins and minimize nonspecific uptake by the reticuloendothelial system (RES), prolonging their blood circulation time [15]. Naturally occurring polysaccharides used as a hydrophilic block, such as alginate, dextran, chitosan, heparin, hyaluronic acid and chondroitin sulfate, are the best candidates for the construction of polymeric micelles due to their well-reported biocompatibility, availability and cost-effectiveness as well as their abundant functional groups, amenable to chemical modifications [16,17,18,19,20].

Grafting of hydrophobic moieties, such as long alkyl chains or hydrophobic polymers, to the backbone of hydrophilic polysaccharides is the most commonly used method for preparation of amphiphilic polymers, and various preparation procedures have already been described in the reported literature [21,22,23]. For example, Yang et al. prepared amphiphilic cholesteryl-grafted sodium alginate using *N*,*N*-dicyclohexylcarbodiimide as a coupling agent and 4-dimethylaminopyridine as the catalyst at room temperature, where the derivatives formed self-aggregates with a size of approximately 136 nm in an aqueous sodium chloride solution [24]. Similarly, de Oliveira Pedro et al. reported the synthesis of amphiphilic *N*-(*N*,*N*-diethylethylamine)-*N*-dodecyl chitosan. The formed *N*-(*N*,*N*-diethylethylamine)-*N*-dodecyl chitosan self-aggregates exhibited a high entrapment efficiency for quercetin of more than 73%, and the release study followed a Fickian diffusion mechanism and controlled releasing process [25].

Belonging to the category of one-pot reactions, multicomponent reactions (MCRs), in which at least three components react to form a single product that retains all or most of the atoms of the starting materials [26], have been emerging as a powerful tool for the synthesis of biologically active compounds because of unique advantages, such as high efficiency, atomic economy, waste reduction, as well as time and energy economy; this is consistent with most of the green chemistry principles of Anastas and Warner [27]. The Ugi four-component reaction (Ugi-4CR) is one of the most famous MCRs reported so far to directly obtain N-acylated α-aminoamides in one step by the reaction of a carboxylic acid, an aldehyde, an amine and an isonitrile [28]. In polymer chemistry, the Ugi-4CR also shows its potential in the preparation of highly functional polymers [28,29,30]. 

Alginates, one of the most naturally abundant anionic polysaccharides extracted from different species of marine brown algae and bacteria, have been extensively investigated and used for many biomedical and pharmaceutical applications, including drug delivery, encapsulation of enzymes and cells as well as wound healing management and tissue regeneration, due to its excellent biocompatibility, biodegradability, non-toxicity, relatively low cost, mild gelation by the addition of divalent cations (Ca^2+^), and the activity of carboxylic and hydroxyl groups [31,32,33,34]. In addition, they have already been granted permission from the U.S. Food and Drug Administration (USFDA) for human use [35]. Alginates are linear copolymers that consist of (1–4)-linked β-D-mannuronic acid (M) and its C-5 epimer α-L-guluronic acid (G), which are arranged in repeating GG (MM) blocks or alternating MG blocks. The content of the M and G residues in the framework and the physical properties, such as the molecular weight of the copolymer, vary with the source and type of alginate [36]. However, alginates with high molecular weights are difficult to degrade through cleavage of glycosidic linkages under physiological conditions [37]. This mainly restricts its applications in drug delivery. It is now well-established that the oxidation of alginate with sodium periodate on the hydroxyl group (-OH) at the C-2 and C-3 positions of the uronic units can enhance its biodegradability and reactivity, providing an interesting opportunity for broadening the biomedical applications of alginate [38,39,40]. However, there are few reports on OSA-based amphiphilic polymer as a smart carrier for drug delivery, especially as a pH-responsive carrier for oral hydrophobic drugs.

Herein, we report a catalyst-free approach for green and efficient preparation of the innovative OSA-based amphiphilic conjugate (Ugi-OSAOcT) through an Ugi-4CR from octylamine (OCA), oxidized sodium alginate (OSA), acetic acid (HAc) and tosylmethyl isocyanide (TOSMIC) in distilled water at room temperature. The chemical structure and thermal property of the Ugi-OSAOcT conjugate were characterized by FTIR, 1H NMR, XRD, EA, GPC and TGA. The influencing factors, including the reaction time, temperature, and feed molar ratio of the reactant on the degree of substitution (DS) were investigated. The self-assembly behavior, particle size, zeta potential, morphology and stability of the micelles in aqueous solutions were evaluated by FM, DLS and TEM. In addition, the in vitro cytotoxicity of the Ugi-OSAOcT conjugates was evaluated by MTT assay. Ibuprofen (IBU), which served as a model poorly water-soluble drug, was incorporated into the Ugi-OSAOcT micelles by dialysis method. The physicochemical characteristics of the IBU-loaded Ugi-OSAOcT micelles and in vitro drug-release behavior under different pH values were also investigated. The results revealed that the amphiphilic Ugi-OSAOcT conjugates could have potential applications for effective entrapment and oral delivery of hydrophobic bioactive compounds.

## 2. Materials and Methods

### 2.1. Materials

Sodium alginate (SA, Mw: 198,000, Mn: 136,000) was obtained from J&K Reagent Technology Co., Ltd. (Beijing, China), and the molecular weight was measured by gel permeation chromatography (GPC). The 3-(4,5-dimethylthiazol-2-yl)-2,5-diphenyltetrazolium bromide (MTT) was purchased from Macklin Biochemical Co., Ltd. (Shanghai, China). Sodium periodate (AR, 99.5%), ethylene glycol (AR, 98%), tosylmethyl isocyanide (TOSMIC, 98%), octylamine (OCA, 99%), acetic acid (HAc, 99.5%), ethanol absolute (AR), methanol (AR), *N*,*N*-dimethylformamide (AR, 99.5%), sodium chloride (AR, 99.5%), model drug ibuprofen (98%) and pyrene as a fluorescence probe were purchased from Aladdin Chemical Reagent Co., Ltd. (Shanghai, China). RAW 264.7 cells were purchased from the Cell Bank of the Chinese Academy of Sciences (Shanghai, China). DMEM medium were obtained from Gibco (Thermo Fisher Scientific, Waltham, MA, USA). Fetal bovine serum (FBS) was supplied by Biological Industries (Rehovot, Israel). The dialysis bag (MWCO 3500) was purchased from Beijing Laibo Runke Biotechnology Co., Ltd. (Beijing, China) for drug release studies. All reagents were of analytical grade and used without further purification. Deionized water was used in all the experiments.

### 2.2. Synthesis of Ugi-OSAOcT Conjugates

#### 2.2.1. Oxidation of Sodium Alginate

OSA was prepared according to the protocol previously reported by Gomez et al. with some modifications [39]. In brief, 4.0 g (20.2 mmol) of sodium alginate (SA) was dissolved in 200 mL of distilled water, the required amount of sodium periodate was added and the mixture was stirred for 24 h under dark conditions at room temperature. The molar ratios of sodium periodate to monomeric unit of SA were 1:10, 3:10 and 5:10, respectively. The reaction was quenched by adding equimolar ethylene glycol to sodium periodate and stirring for 0.5 h. Then, 500 mL of ethanol and 1.50 g (0.025 mol) of NaCl was added to the mixture. The precipitate was collected by vacuum filtration, re-dissolved in deionized water and dialyzed against distilled water using a dialysis bag (MWCO 3500 Da) for 3 days and, finally, lyophilized to obtain oxidized sodium alginate (OSA), named as OSA_10_, OSA_30_ and OSA_50_, respectively. In this paper, the subscripts in OSA_10_, OSA_30_ and OSA_50_ mean the theoretical oxidation degree of OSA.

The degree of oxidation of OSA was followed by determining the concentration of unreacted periodate by iodometry, according to the previous method [39,40]. Based on titration results, the degree of oxidation of OSA_10_, OSA_30_ and OSA_50_ can be calculated as 9.51%, 27.76% and 44.25% (Table 1), respectively.

#### 2.2.2. OSA Modification with OCA

Ugi-OSAOcT was synthesized via an Ugi-4CR according to the procedure described by Yan et al. with some modifications [41]. Briefly, 2.0 g (10.0 mmol) of OSA (OSA_10_, OSA_30_ and OSA_50_) was first dissolved in 200 mL of deionized water (1.0%, m/v), and 4.0 mL (24.0 mmol) of OCA was added under mechanical stirring at room temperature for 30 min. Then, 1.71 mL (30.0 mmol) HAc and 6.44 g (33.0 mmol) of TOSMIC dissolved in 5.0 mL of DMF was added into the reaction solution and mechanically stirred at room temperature for 12 h. Subsequently, volume of absolute ethanol five times greater was added to the mixture to precipitate product. The precipitate was collected by centrifugation, re-dissolved in deionized water and dialyzed against distilled water using a dialysis bag (MWCO 3500 Da) for 3 days. Subsequently they were lyophilized to obtain the target Ugi-OSAOcT, named as Ugi-OSA_10_OcT, Ugi-OSA_30_OcT and Ugi-OSA_50_OcT, respectively. 

### 2.3. Characterizations of Ugi-OSAOcT Conjugates

#### 2.3.1. FTIR and ^1^H NMR Spectroscopy

The structure of the Ugi-OSAOcT conjugates was characterized by FTIR and ^1^H NMR spectroscopy. In detail, the FTIR spectra of the sample were recorded on a Nicolet-6700 (Thermo Scientific, Waltham, MA, USA) with KBr pellets in the range of wavenumbers between 4000 and 400 cm^−1^ for 64 scans with a spectral resolution of 2.0 cm^−1^. The ^1^H NMR spectra was recorded at 25 °C using an ULTRASHIELD 400 PLUS spectrometer (Bruker, Fällanden, Switzerland) operating at 400 MHz with deuterated water (D_2_O) as a solvent and tetramethylsilane (TMS) as an internal standard; the concentration of the sample was approximately 8.0–10.0 mg/mL. 

#### 2.3.2. X-ray Diffraction Analysis

The XRD pattern was performed over a 2θ range from 5° to 60° on an AXS/D8 X-ray diffractometer (Bruker, Cambridge, UK) equipped with graphite monochromatized high-intensity Cu-Kα radiation (λ = 0.154 nm, 40 kV, 100 mA). The scan rate was 2°/min. 

#### 2.3.3. Thermogravimetric Analysis

Thermal stability was studied by thermogravimetric analysis (TGA). In detail, a 9.0–10.0 mg sample was weighed and placed on a 449F3 thermogravimetric analyzer (TA Instrument, New Castle, DE, USA) with aluminum crucibles as a sample holder. Then, the temperature was increased from 20 °C to 800 °C at a heating rate of 20 °C/min under the protection of high purity nitrogen (100 mL/min, 0.04 MPa). 

#### 2.3.4. Measurement of Degree of Substitution

The degree of substitution (DS, %), defined as the number of OCA molecules per 100 sugar residues of OSA, was quantified by elemental analysis according to the method described in the existing literature [41]. The elemental contents of *C*, *H* and *N* (% m/m) of the sample were determined using Elementar’s vario EL Cube (Elementar, Germany). The DS was calculated based on the *C* and *N* contents (% m/m) according to the following Equation (1):

(1) $$\mathrm{DS}=\frac{6\alpha M_{C}}{2M_{N}-19\alpha M_{C}}\times 100\%$$

where *α*, *M_C_* and *M_N_* erefer to the *N/C* content ratio, relative atomic mass of *C* and relative atomic mass of *N*, respectively.

#### 2.3.5. Gel Permeation Chromatography Analysis

A GPC (Waters e2695, Milford, MA, USA) equipped with an Ultrahydrogel^TM^120 (7.8 × 300 mm^2^) column was applied to measure the weight-average molecular weight (Mw), number-average molecular weight (Mn) and polydispersity index(PDI) of the Ugi-OSAOcT conjugates. The concentration of the sample was 1 mg/mL, and 0.05% sodium azide was used as the mobile phase with a flow rate of 0.6 mL/min at 40 °C.

### 2.4. Measurement of Critical Micelle Concentration (CMC)

The CMC of the Ugi-OSAOcT conjugates in 0.05 mol/L of NaCl aqueous solution was determined using pyrene as a fluorescent probe on a F7000 fluorescence spectrophotometer (Hitachi, Honshu, Japan) [42]. Briefly, a series of concentration ranges of 5.0 × 10^−4^–2.0 mg/mL of the Ugi-OSAOcT solutions were prepared by adding 0.05 mol/L of NaCl solution and 10 μL of pyrene (1.0 × 10^−3^ mol/L in methanol) to the aforementioned solution (10.0 mL), respectively. All solutions were sonicated in ultrasonic bath and left to evaporate overnight at 25 °C. Pyrene was excited at 335 nm, and its emission spectra was recorded in the range of 350–600 nm at an integration time of 1 s with a slit width of 2.5 nm. The ratio of fluorescence intensity at 373 and 384 nm (I_1_/I_3_) was calculated and plotted against the logarithmic concentration of the corresponding samples to determine the CMC value of the conjugates.

### 2.5. Preparation of Blank Ugi-OSAOcT Micelles

The blank Ugi-OSAOcT micelles were prepared by directly dispersing the Ugi-OSAOcT conjugates in deionized water, followed by sonication to facilitate aqueous dispersion and self-assembly. Briefly, the Ugi-OSAOcT conjugates were dissolved in distilled water to make a concentration of 1 mg/mL under gentle stirring at 25 °C for 6 h. The solution was then sonicated for 4 min by a probe sonicator at 120 W and repeated 3 times in order to guarantee optically clear dispersion. The sonication pulse was turned on 2 s with a waiting time of 4 s between pulses. Finally, the micellar solution was further filtered through a 0.45 μm syringe filter and stored at 4 °C.

### 2.6. Characterization of Polymeric Micelles

#### 2.6.1. Dynamic Light Scattering (DLS)

The particle size, polydispersity index (PDI) and zeta potential of the Ugi-OSAOcT micelles in aqueous solution were measured by the DLS experiments using the Zetasizer Nano ZS90 (Malvern, Worcestershire, UK) at 25 °C with an argon laser (He-Ne) light wavelength of 633 nm at a 90° scattering angle. The solution of conjugates was maintained at a concentration of 1 mg/mL and was passed through a 0.45 μm microfilter before DLS measurement.

#### 2.6.2. Transmission Electron Microscopy (TEM)

The morphologies of the Ugi-OSAOcT micelles were observed on a TEM instrument (JEM2100, JEOL Co., Tokyo, Japan) operated at an acceleration voltage of 200 kV. Prior to obtaining the images, several drops of micelles solution were dropped on a carbon-coated copper grid, stained with 2% (*w*/*v*) phosphotungstic acid for 20 s and then left to dry at room temperature for 30 min.

#### 2.6.3. Storage Stability of Blank Ugi-OSAOcT Micelles

To evaluate the storage stability of the prepared micelles, the blank Ugi-OSAOcT micelles (1 mg/mL) were dispersed in PBS (pH 7.4) and incubated at 25 °C for 0, 1, 2, 4, 6, 8, 10 and 15 days. The mean size, PDI and zeta potential of the micelles were monitored by DLS. All measurements were repeated in triplicate to ensure reproducibility of the results.

### 2.7. Preparation of IBU-Loaded Self-Assembled Nanoparticles

The IBU-loaded Ugi-OSAOcT nanoparticles were prepared by an ultrasound dialysis method [43]. Typically, 10.0 mg of Ugi-OSAOcT conjugates were dissolved in 10 mL distilled water. Different volumes of IBU methanol solution (0.5 mg/mL) were then added dropwise to the solution under gentle stirring for 2 h at room temperature and further sonicated for 10 min by probe-type ultra sonicator. Subsequently, the whole solution was transferred to a dialysis bag (MWCO 3500 Da) and dialyzed against distilled water for 48 h to remove the organic solvents and free IBU. Distilled water was exchanged at 4 h intervals during the dialysis procedure. After dialysis, the final solutions were centrifuged at 8000 rpm for 20 min, and the supernatant was filtered through a 0.45 µm syringe filter to remove the insoluble IBU and then lyophilized for 48 h to obtain micelle powders. 

To determine the IBU-loading contents, a known amount of freeze-dried IBU-loaded nanoparticles was dissolved in methanol (1 mL), sonicated for 30 min and filtered through a 0.45 μm syringe filter. The IBU concentration was measured by using a UV-vis spectrophotometer (U-3900, Hitachi, Japan) at 222 nm based on the standard calibration curve obtained from free IBU in methanol. The encapsulation efficiency (%EE) and drug loading (%DL) were calculated according to Equations (2) and (3), as follows, respectively:

(2) $$\%\mathrm{EE}=\frac{Weight\;of\;IBU\;loaded\;in\;micelles}{Weight\;of\;feeding\;IBU}\times 100$$

(3) $$\%\mathrm{DL}=\frac{Weight\;of\;IBU\;loaded\;in\;micelles}{Total\;Weight\;of\;micelles}\times 100$$

### 2.8. In Vitro Drug Release Studies

To evaluate the suitability of the Ugi-OSAOcT nanoparticle for a variety of applications, the in vitro release behavior of IBU from an IBU-loaded Ugi-OSAOcT nanoparticle was investigated by a common dialysis method in PBS buffer media with different pH values (0.1 M, pH 1.2, 5.0 and 7.4) at 37 °C. The PBS media with pH values of 1.2 and 7.4 were respectively used as the simulated gastric fluid and simulated intestinal fluid. Tween 80 (0.5%, *w*/*v*) used as a surfactant was added to the PBS media to maintain the sink conditions [44]. Briefly, 1.0 mL of IBU-loaded Ugi-OSAOcT nanoparticle solution was placed in dialysis bag (MWCO 3500 Da) and then immersed completely in 30 mL of release media. The whole release system was incubated in a thermostat water bath and shaken at 100 rpm at 37 °C. At the predetermined time points (0.5, 1, 3, 5, 7, 9, 12, 15, 18, 24, 36 and 48 h), 3 mL of the release media was withdrawn, and the same volume of fresh release media were then added to maintain a constant volume. The released amounts of IBU were measured by a UV–vis absorption experiment at a wavelength of 222 nm. For comparison, similar release experiments were performed with the same amount of free IBU as found in the IBU-loaded nanoparticle. Before the UV absorption experiments, calibration curves of IBU in the PBS-containing Tween80 (0.5%, *w*/*v*) were plotted. All experiments were performed in triplicate. The percentage of cumulative drug release was calculated from Equation (4) as follows:

(4) $$E_{r}\left(\%\right)=\frac{3\sum_{1}^{n-1}C_{i}+30C_{n}}{W_{loadedIBU}}\times 100$$

where *C_i_* and *C_n_* refer to the concentrations of IBU extracted from release media at *i* and *n* time, respectively, and *n* is the number of times the sample solution was withdrawn. *W_loaded IBU_* is the weight of IBU previously loaded in the Ugi-OSAOcT nanoparticles.

### 2.9. In Vitro Cytotoxicity Assays

The cytotoxicity of the Ugi-OSAOcT conjugates, OSA and SA (control) against RAW 264.7 cells were evaluated by MTT assay [45,46]. In detail, RAW 264.7 cells were seeded into 96 well plates at a density of 4.0 × 10^3^ cells/well in the incubator (37 °C, 5% CO_2_) for 24 h. The culture medium was replaced with 100 μL of the sample solutions containing a series of concentrations (0, 200, 400, 600, 800 and 1000 μg/mL) in DMEM. Following incubation for 48 h, the culture medium was then replaced with fresh DMEM (100 μL), followed by an addition of 20 μL of MTT solution in PBS (5 mg/mL), and incubated for an additional 4 h at 37 °C in CO_2_. The MTT-containing medium was then removed, and 100 μL of DMSO was added to each well to solubilize the formed formazan crystal with gentle agitation for 10 min. The absorbance of the solution was measured at 570 nm with a Multiskan MK3 microplate reader (Thermo, Waltham, MA, USA). All experiments for this study were performed in triplicate, and the cell viability (%) was calculated from Equation (5) as follows:

(5) $$\%Cell\;viablilty=\frac{Ab_{S570\mathrm{nm}}sample-Ab_{S570\mathrm{nm}}blank}{Ab_{S570\mathrm{nm}}control-Ab_{S570\mathrm{nm}}blank}\times 100$$

where *Abs*_570nm_ blank refers to the absorbance of DMSO at 570 nm without cells. *Abs*_570nm_ sample and *Abs*_570nm_ control refer to the absorbance at 570 nm in the presence and in the absence of treatment samples, respectively. 

### 2.10. Statistical Analysis

All data are presented as the mean value ± standard deviation. A single factor ANOVA was performed by SPSS software to analyze the variables, and a value of *p* < 0.05 was considered statistically significant.

## 3. Results and Discussion

### 3.1. Synthesis and Characterization of Ugi-OSAOcT Conjugates

**Figure 1:**
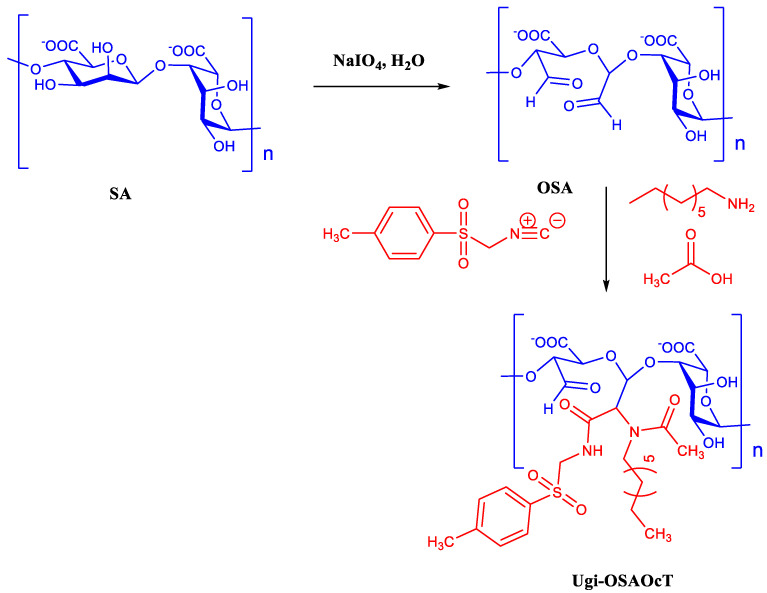
Schematic illustration of synthesis routes of Ugi-OSAOcT.

**Figure 2:**
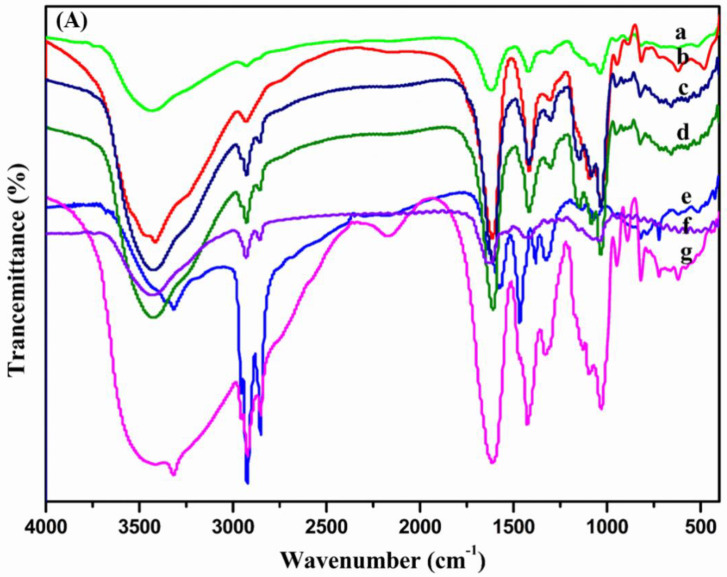

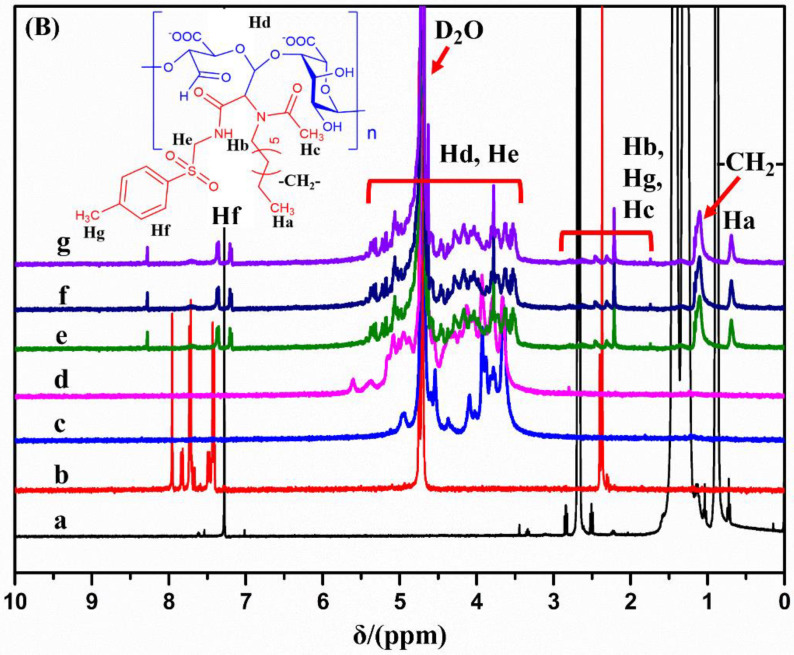
(**A**) FTIR spectra of (a) SA, (b) OSA, (c) Ugi-OSA_10_OcT, (d) Ugi-OSA_30_OcT, (e) OCA, (f) Ugi-OSA_50_OcT and (g) OCA+OSA mixture; (**B**) ^1^H NMR spectra of (a) OCA (CDCl_3_), (b)TOSMIC (D_2_O), (c)SA (D_2_O), (d)OSA (D_2_O), (e)Ugi-OSA_10_OcT (D_2_O), (f) Ugi-OSA_30_OcT (D_2_O) and (g) Ugi-OSA_50_OcT (D_2_O).

The Ugi-OSAOcT conjugates with various DS were straightforwardly synthesized by grafting OCA to the OSA backbone via an Ugi-4CR, and the detailed synthetic scheme of the Ugi-OSAOcT conjugate was presented in Figure 1. Water, a non-toxic and safe solvent, can significantly reduce the impact of the process on the environment of the reaction. Accordingly, we envisioned that the utilization of an Ugi-4CR would provide a rapid and effective synthetic way to constructi novel amphiphilic polymers, due to the advantages of simplicity, atom-economy and good yields. Successful synthesis of the conjugates was confirmed by FTIR and ^1^H NMR, as shown in Figure 2A,B, respectively.

The FTIR spectra of SA, OSA, OCA and the Ugi-OSAOcT conjugates were represented in Figure 2A. The strong and broad band at 4000–3000 cm^−1^ was assigned to the O-H stretching vibration of polysaccharide, and the band at 2925 cm^−1^ was attributed to the C-H stretching vibration of the polysaccharide structure [47]. The characteristic absorption peak of SA at 1621 cm^−1^ and 1419 cm^−1^ belonged to the asymmetric and symmetric stretching vibrations of -COO-, respectively [48]. The band at 1100–890 cm^−1^ was attributed to ether groups of the polysaccharide skeleton (C-O-C stretching) [49]. However, OSA’s characteristic band at 1734 cm^−1^ was too weak and hard to be detected due to the formation of hemiacetal by the free aldehyde groups [50]. The characteristic band of OCA was also identified at 3300 cm^−1^ (N-H stretching), at 2925 cm^−1^ (-CH_2_ stretching) and at 2842 cm^−1^ (-CH_3_ stretching). After conjugation of OCA to the OSA backbone, the abovementioned typical bands of OSA appeared in the Ugi-OSAOcT conjugate with various DS spectra. In addition, the characteristic absorption peaks corresponding to OCA (2925 cm^−1^ and 2842 cm^−1^) were both observed, which confirmed successful introduction of OCA in the OSA skeleton [25]. Furthermore, it was possible to compare the Ugi-OSAOcT conjugate with its physical mixture, where the amino group of OCA is well observed, further supporting successful synthesis of the Ugi-OSAOcT conjugates.

The structures of the Ugi-OSAOcT conjugates were further confirmed by ^1^H NMR. From Figure 2B, the typical peaks of SA were found at δ = 3.5–5.0 ppm, assigned to the methylene of uronic acid for SA [48]. In comparison with SA, the new signals of OSA at about δ = 5.35, 5.02 and 4.30 ppm confirmed the successful oxidation of SA with sodium periodate, which were attributed to a hemiacetalic proton generated formaldehyde groups with hydroxyl groups present on the adjacent uronic acid subunits on the OSA [39]. On the Ugi-OSAOcT conjugate spectra, the peaks at δ = 0.8, 1.1–1.5 and 2.0–3.0 ppm were attributed to the methyl group (-CH_2_-CH_3_), methylene (-(CH_2_)_6_-CH_2_-NH-) and methylene (-(CH_2_)_6_-CH_2_-NH-) of OCA, respectively. The characteristic peaks at δ = 2.30 and 7.0–8.0 ppm were attributed to the methyl group (Ph-CH_3_) and its phenyl protons (Ph-H) of TOSMIC, respectively. Therefore, the presence of the typical peaks of OSA, OCA and TOSMIC in the Ugi-OSAOcT conjugate spectra further confirmed successful synthesis of the Ugi-OSAOcT conjugates via the Ugi-4CR [25]. 

**Figure 3:**
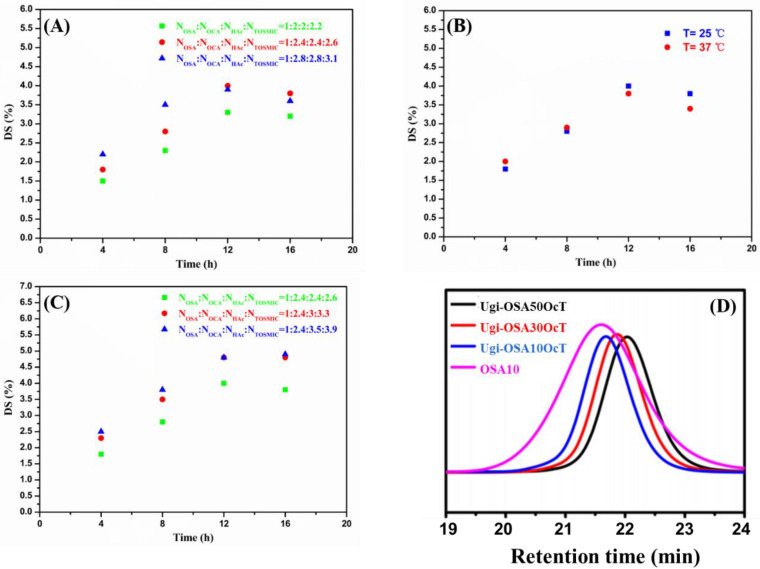
(**A**–**C**) DS (%), as a function of reaction time after coupling of OCA to OSA10 varying: (**A**) molar ratio of OCA to OSA at 25 °C; (**B**) temperature (25 °C and 37 °C) using the molar ratio of 1:2.4:2.4:2.6 (NOSA10:NOCA:NHAc:NTOSMIC); and (**C**) amount of HAc at constant molar ratio of 1:2.4 (NOSA10:NOCA) and 25 °C. (**D**) GPC traces of OSA_10_ and Ugi-OSAOcT conjugates.

The DS of the Ugi-OSAOcT conjugates was calculated by the element contents of C and N obtained from EA data. The optimum reaction parameters for coupling of the primary amines to OSA via Ugi-4CR were found through a series of experiments (see Table 2). In all the optimization experiments, the influence of the feed molar ratio of OSA to OCA, temperature and amounts of HAc using the same batch of OSA_10_ on DS were investigated. As SA was oxidized by sodium periodate to obtain OSA with a dialdehyde structure, all experiments were carried out on the basis of a molar ratio of OSA to OCA (1:2), in order to obtain a high DS. As shown in Figure 3A–C, the DS of the Ugi-OSAOcT conjugates increased with an increasing reaction time for all combinations of the reaction parameters and reached the plateau DS values at 12 h.

The DS could be increased by increasing the amounts of OCA. As shown in Figure 3A, the feed molar ratio of OSA to OCA increased from 1:2 to 1:2.4, corresponding to an increase in the DS from 3.3% to 4.0%. It then continued to increase to 1:2.8 as the DS slightly decreased to 3.9%. The reason for this may be that an increase in the feed amount of OCA will increase the alkalinity of the solution, making OSA more easily decomposed and degraded, resulting in a decrease in DS. Whereas, increasing the temperature from 25 °C to 37 °C had a definite effect on the DS (Figure 3B), indicating that a temperature beyond 25 °C resulted in the degradation or hydrolysis of isocyanide. Therefore, the most appropriate temperature proved to be around 25 °C. In addition, the DS increased from 4.0% to 4.9% with an increase in the molar ratio of OSA to HAc from 1:2.4 to 1:3.5, suggesting that the DS could be substantially increased by increasing the amount of HAc (Figure 3C). This may be attributed to the fact that the HAc was conducive to the protonation of imine intermediates and promoted progress in the Ugi-4CR.

Overall, the optimum reaction conditions for synthesizing a high DS of the Ugi-OSAOcT conjugate was obtained, which were a reaction time of 12 h, a reaction temperature of 25 °C and a molar ratio of 1:2.4:3:3.3 (OSA:OCA:HAc:TOSMIC), respectively, and a series of Ugi-OSAOcT conjugates with a high DS were prepared using OSA with different oxidation degrees. As shown in Table 1, the DS of the Ugi-OSAOcT conjugates varied from 4.8% to 24.3% with an increase in the oxidation degree of OSA from 9.51% to 44.25%. In addition, the molecular weight of the Ugi-OSAOcT conjugates decreased with an increase in the oxidation degree of OSA (Figure 3D). These results are consistent with previous findings in other reports [51].

**Figure 4:**
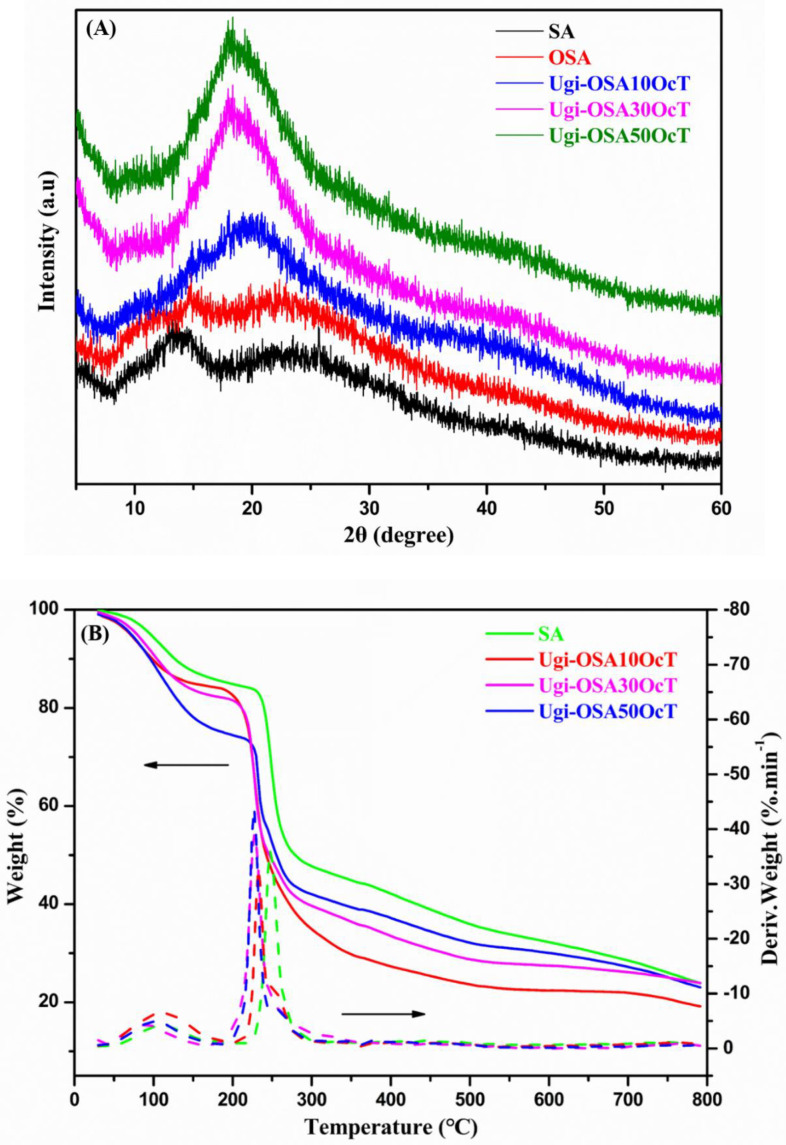
(**A**) XRD patterns of SA, OSA and Ugi-OSAOcT conjugates. (**B**) TGA and DTG curves of SA and Ugi-OSAOcT conjugates.

XRD is the most direct and effective method for analyzing the crystallinity of the polymers, which has great influence on drug incorporation, micelle stability, and drug release. Importantly, the research of Zhang et al. has revealed that an amorphous structure was favorable for drug loading [52]. As shown in Figure 4A, the XRD patterns of SA and OSA showed diffraction peaks at 2θ = 14.8, 22.3° and 2θ = 15.1°, 22.4°, respectively, suggesting that the hydrated crystalline structure resulted from the intramolecular hydrogen bonds of SA and OSA. For the Ugi-OSAOcT conjugates, a typical diffraction peak at 2θ = 20° was observed, indicating that the Ugi-OSAOcT conjugates became amorphous due to the introduction of hydrophobic OCA units in the OSA block and destruction of inter- and intramolecular hydrogen bonds [53]. These results are consistent with those reported by Prabaharan et al. and Zong et al. [54,55].

The thermal stability of the Ugi-OSAOcT conjugates with different DS was evaluated by TGA and DTG. The TGA curves were shown in Figure 4B. SA and the Ugi-OSAOcT conjugates displayed similar thermal degradation behaviors, revealing two notable weight loss stages. The first one began at 80–120 °C, which was attributed to the evaporation of the physically adsorbed and encapsulated water in the samples [56,57]. The second weight loss stage took place between 200–300 °C, corresponding to the decomposition of the Ugi-OSAOcT conjugates’ molecular skeletons [58]. It can be also observed that the initiatial decomposition temperature of the Ugi-OSAOcT conjugates was independent of their DS, which was lower than that of SA (248 °C), indicating that the thermal stability of the Ugi-OSAOcT conjugates decreased. This result could be attributed to the introduction of hydrophobic groups that reduced the carboxyl groups of polymers, thus destroying the intramolecular hydrogen bonds of SA, which was consistent with the XRD analysis. 

### 3.2. Self-Assembly Behavior of Ugi-OSAOcT Conjugate

**Figure 5:**
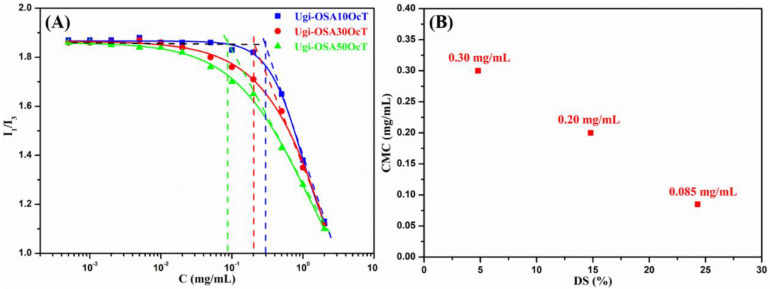
(**A**) Plots of pyrene fluorescence intensity ratio (I_1_/I_3_) vs. the concentration of Ugi-OSAOcT in 0.05 mol/L aqueous NaCl solution at 25 °C; (**B**) changes in the CMC value of Ugi-OSAOcT micelles in 0.05 mol/L aqueous NaCl solution at 25 °C as a function of DS.

The self-assembly behavior of the amphiphilic conjugate was first evaluated by measuring the CMC with a pyrene fluorescence probe technique. Pyrene displays five characteristic emission peaks in the range of 370–400 nm. The peak intensity is sensitive to the micro-environmental polarity surrounding the pyrene molecules. As illustrated in Figure 5A, the ratio of pyrene fluorescence intensities emitted at 373 and 384 nm (I_1_/I_3_) remained almost unchanged at low concentrations of the amphiphilic conjugate and decreased sharply once the amphiphilic conjugate concentration reached the CMC, indicating that the micelles were formed, and the pyrene probe molecules were encapsulated, in the hydrophobic interior of the micelles. The intensity ratio of I_1_/I_3_ was plotted as a function of the logarithm of conjugate concentration to obtain the CMC value. The CMC values of Ugi-OSA_10_OcT, Ugi-OSA_30_OcT and Ugi-OSA_50_OcT were found to be 0.30, 0.20 and 0.085 mg/mL, respectively, which was significantly lower than that of the surfactant for sodium dodecyl sulfate (CMC = 2.3 mg/mL) [59]. It can be observed from Figure 5B that the CMC value of the Ugi-OSAOcT conjugates decreased with an increase in the DS. This was possibly ascribed to the introduction of an OCA unit that provided more hydrophobicity to the Ugi-OSAOcT conjugates, thus exhibiting a higher tendency for self-assembly in an aqueous solution, which was similar to the previously reported amphiphilic block copolymers [60]. Moreover, an amphiphilic conjugate with a lower CMC value could show a higher thermodynamic stability and retain intact micellar structures even under a high dilution condition, achieving the aim of a long blood circulation time [61,62].

### 3.3. Preparation and Characterization of Ugi-OSAOcT Micelles

**Figure 6:**
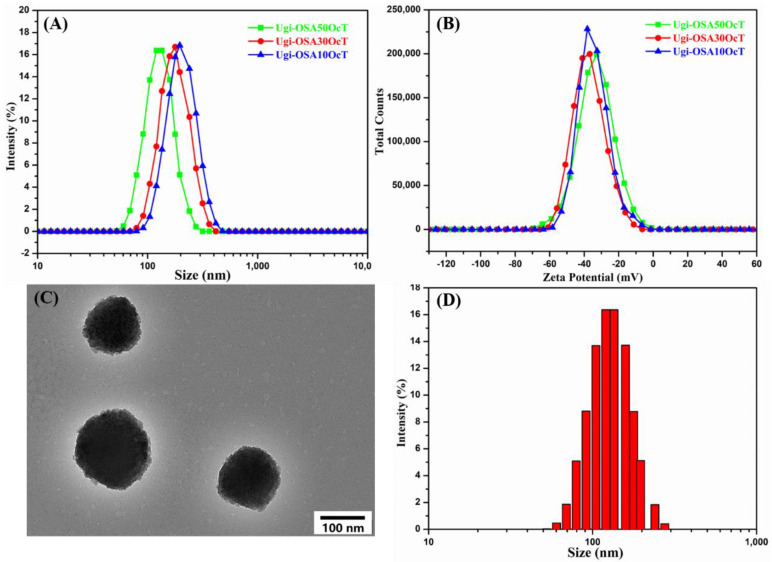
(**A**) Size distribution and (**B**) zeta potential determined with DLS for the blank Ugi-OSAOcT micelles with different DS at 25 °C; (**C**) TEM; and (**D**) size distribution for the blank Ugi-OSA_50_OcT micelles at 25 °C. The mass concentration of Ugi-OSAOcT conjugates was 1.0 mg/mL. Data are presented as mean ± SD (*n* = 3).

An ultrasonic is a commonly used approach to induce the self-assembly of the amphiphilic Ugi-OSAOcT conjugates in order to achieve the formation of micelles in an aqueous solution. The particle size and zeta potential, characterized by DLS, are crucial parameters for nanoparticle evaluations. Table 3 summarizes the particle size and zeta potential of Ugi-OSA_10_OcT, Ugi-OSA_30_OcT and Ugi-OSA_50_OcT micelles (1.0 mg/mL). As shown in Figure 6A, when the DS of the Ugi-OSAOcT conjugates increased from 4.8 to 24.3%, the particle size of the micelles decreased from 196.5 ± 3.8 to 135.7 ± 2.4 nm, suggesting that an increase in hydrophobic block content enhanced the hydrophobic interaction between hydrophobic groups, resulting in a more compact cores and a smaller particle size. Zeta potential is one of the important factors for maintaining the stability of micelles. In general, micelles with an absolute value of zeta potential greater than 30 mV have good stability due to electrostatic repulsion [63,64]. Figure 6B showed that the zeta potential of the Ugi-OSAOcT micelles (1.0 mg/mL) was around −35 mV, indicating the presence of multiple ionized carboxyl groups (-COO^−^) on the micelles’ surfaces, favoring long-term storage stability and long circulation in the body, due to strong electrostatic repulsion between micelles.

A TEM image showed the morphologies of the self-assembled Ugi-OSA_50_OcT micelles (1.0 mg/mL). As shown in Figure 6C, all micelles were almost spherical with a size ranging from 90–110 nm and were well-dispersed with no aggregation. The particle size obtained from TEM was found to be smaller than that obtained by DLS measurement (135.7 ± 2.4 nm), as shown in Figure 6D. This phenomenon was ascribed to shrinkage of the micelles caused by water evaporation during air drying during the TEM analysis, while the DLS measurements were carried out in an aqueous medium, where the micelles were in swollen form [65].

**Figure 7:**
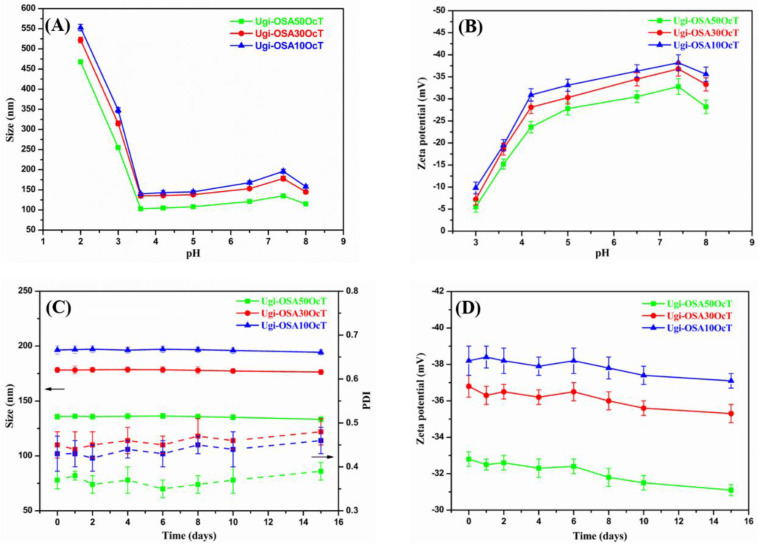
Influence of pH on: (**A**) the size; (**B**) zeta potential of Ugi-OSAOcT micelles with different DSat 25 °C; (**C**) the size, PDI and (**D**) zeta potential of Ugi-OSAOcT micelles over time at 25 °C in PBS (pH 7.4). The mass concentration of Ugi-OSAOcT conjugates was 1.0 mg/mL. Data are presented as mean ± SD (*n* = 3).

Figure 7A,B illustrates the influence of pH on the particle size and zeta potential of the Ugi-OSAOcT micelles. When the pH decreased from 5.0 to 3.6, the zeta potential increased promptly from −27.8–−33.1 mV to −15.2–−19.5 mV. Nevertheless, no significant changes in the particle size of the micelles were observed. This may be attributed to the fact that the beginning of protonation of the carboxylic acid groups reduced electrostatic repulsion among the polymer chains and increased the hydrophobicity of micelles, resulting in a decrease in the swelling capacity of the micelles. When the pH increased from 5.0 to 7.4, the particle size of the Ugi-OSA_10_OcT, Ugi-OSA_30_OcT and Ugi-OSA_50_OcT micelles increased from 145,138 and 108 nm to 196,178 and 135 nm, respectively, suggesting that an increase in deprotonation of the carboxylic acid groups enhanced electrostatic repulsion among the polymer chains and hydrophilicity of the micelles, giving rise to the swelling behavior of the micelles, which could be further supported by the decrease in zeta potential from −27.8–−33.1 mV to −32.8–−38.2 mV. A similar swelling behavior of the polymeric nanoparticles responding to pH changes has been reported elsewhere [66].

The long-term stability of the Ugi-OSAOcT micelles (1.0 mg/mL) of up to 15 days was also performed in PBS (pH 7.4) at 25 °C by measuring the particle size, PDI and zeta potential. As shown in Figure 7C,D, the particle size and zeta potential of all micelles did not significantly change during the first six days. With the extension of storage time to 15 days, the particle size and absolute value of the zeta potential decreased slightly due, to a certain extent, the degradation of the polymer micelles, indicating good long-term stability in PBS (pH 7.4), which is in line with the fact that the micelles generally have good stability under neutral conditions [67].

### 3.4. Preparation and Characterization of IBU-Loaded Ugi-OSAOcT Micelles

**Figure 8:**
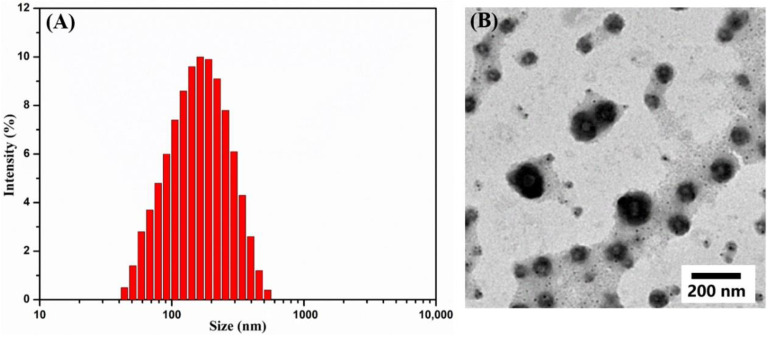
(**A**) Size distribution determined with DLS and (**B**) TEM for IBU-loaded Ugi-OSA_50_OcT micelles at 25 °C. The feeding mass ratio of IBU to Ugi-OSA_50_OcT was 3:10.

The IBU-loaded Ugi-OSAOcT micelles were obtained by self-assembly and purified by dialysis in order to remove the organic solvent. A series of IBU-loaded Ugi-OSAOcT micelles with various drug feeding ratios were prepared, and their characteristics were shown in Table 4. It was found that the sizes of the IBU-loaded micelles increased as the loading content increased, suggesting that the IBU molecules were successfully encapsulated into the hydrophobic inner cores, and the encapsulated IBU molecules increased the size of the Ugi-OSAOcT micelles. The DL and EE of the IBU-loaded Ugi-OSA_50_OcT micelles increased with an increase in the drug feeding ratio. However, when the feeding mass ratio of IBU to Ugi-OSA_50_OcT increased from 3:10 to 5:10, the EE reduced from 57.2 ± 1.3% to 52.4 ± 1.5%, suggesting that the EE reached a maximum value at the mass ratio of 3:10 (IBU:Ugi-OSA_50_OcT). This upward trend in DL and EE could be explained by the enhanced hydrophobic interaction between hydrophobic groups and IBU with the increase in hydrophobic IBU feeding ratio. When the drug content in the micelles reached saturation, severe precipitation occurred with a further increase in the IBU feeding ratio, leading to the decrease in EE. In addition, the size of IBU-loaded Ugi-OSA_50_OcT micelles remained less than 200 nm, which was very crucial for avoiding reticuloendothelial system (RES), as shown in Figure 8A [68,69]. As can be seen in Figure 8B, the TEM image of the IBU-loaded Ugi-OSA_50_OcT micelles also indicated a spherical morphology.

When the feeding mass ratio of the IBU to Ugi-OSAOcT was maintained at 3:10, the size of the IBU-loaded Ugi-OSAOcT micelles displayed a slight decrease with the rise in DS from 210.8 ± 5.2 nm (Ugi-OSA_10_OcT) to 198.6 ± 4.8 nm (Ugi-OSA_30_OcT) and further to 160.3 ± 5.7 nm (Ugi-OSA_50_OcT). This tendency of the particle size was in agreement with that of the blank Ugi-OSAOcT micelles in terms of DS. In addition, the DL of the Ugi-OSA_10_OcT, Ugi-OSA_30_OcT and Ugi-OSA_50_OcT micelles were 10.9 ± 0.4%, 13.2 ± 0.5% and 14.6 ± 0.3%, respectively, corresponding to EE values of 40.8 ± 1.6%, 50.6 ± 1.8% and 57.2 ± 1.3%, respectively. Obviously, the DS influenced both the DL and EE of the IBU-loaded Ugi-OSAOcT micelles. A possible reason is that more hydrophobic OCA groups grafted on the hydrophilic mainchain enhanced the hydrophobic association between the hydrophobic chain, which improved the hydrophobic affinity with the hydrophobic IBU molecules, thereby providing more available sites for hydrophobic interaction with IBU molecules in the inner cores, and thus resulting in an increase in EE and DL. This is in accordance with the results from study by Yokoyama et al. [70].

### 3.5. In Vitro Release of IBU from Ugi-OSAOcT Micelles

**Figure 9:**
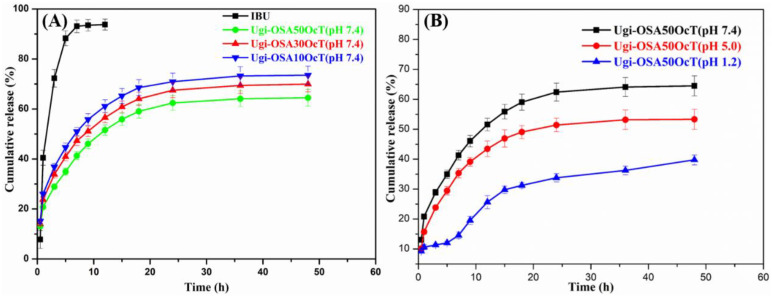
In vitro release profile of: (**A**) free IBU and loaded IBU from Ugi-OSAOcT micelles with different DS at 37 °C in PBS (pH 7.4) containing Tween 80 (0.5% *w*/*v*) and (**B**) loaded IBU from Ugi-OSA_50_OcT micelles at 37 °C in PBS containing Tween 80 (0.5% *w*/*v*) at different pH values. Data are expressed as mean ± SD (*n* = 3).

To investigate the potential utilization of the Ugi-OSAOcT micelles as oral drug carriers, in vitro drug release of IBU from micelles was explored in PBS (pH 7.4 and 5.0) at 37 °C. As shown in Figure 9A, IBU release from micelles occurred in a sustained release pattern compared with that of free IBU, which was found to release 93% within 7 h, confirming that IBU molecules were well encapsulated in the inner core of micelles. The cumulative release amount of IBU from Ugi-OSA_10_OcT, Ugi-OSA_30_OcT and Ugi-OSA_50_OcT was 73.2%, 67.2% and 63.1% within 48 h, respectively, displaying a slight decrease with an increasing DS. A possible reason is that the Ugi-OSAOcT micelles with a high DS could form a more compact hydrophobic core and exhibit a higher affinity with IBU molecules, which reduced the rate of drug diffusion from the micellar core. Therefore, it can be considered as a potential strategy to fine-tune the DS of conjugates for better control of the drug release, thereby retaining more drugs in polymeric micelles during their in vivo transport before reaching target tissue/cells, which provides a higher therapeutic efficacy [71].

The IBU loaded micelles also showed a sustained and pH-dependent drug release manner. As shown in Figure 9B, about 39.8% and 54.1% of IBU were released, respectively, from the Ugi-OSA_50_OcT micelles in PBS (pH = 1.2) and PBS (pH = 5.0), while 63.1% of IBU was released in PBS (pH = 7.4). The results showed slower release behavior at a lower pH. In acidic conditions (pH = 1.2 and 5.0), the weak acid groups (-COOH) in the Ugi-OSA_50_OcT backbone were protonated, and the surface charge of the micelles gradually decreased, which could enhance the hydrophobicity of the micelles and reduce the electrostatic repulsion, thus forming a tighter core-shell structure to make the IBU release more slowly. In actual drug therapy, the prolongation of release time and ideal control performance is beneficial for improving the drug efficacy and drug utilization. In a short period of time after taking the drug, the blood concentration of the drug in the matrix could reach the effective concentration relatively quickly, and then, the concentration could be maintained for a longer period of time, thereby achieving the purpose of sustained release. Moreover, the protonated Ugi-OSA50OcT backbone at a lower pH (simulated gastric fluid) was conducive for protecting the drug from digestion. Consequently, the Ugi-OSA_50_OcT micelles were suitable for oral drug administration [72]. Conversely, the release rates of the IBU in PBS (pH = 7.4) showed a faster and greater release behavior, which made it a potential carrier for liposoluble nutraceuticals, such as IBU with controlled release in gastrointestinal fluid.

### 3.6. In Vitro Cytotoxicity of Ugi-OSAOcT Conjugates

**Figure 10:**
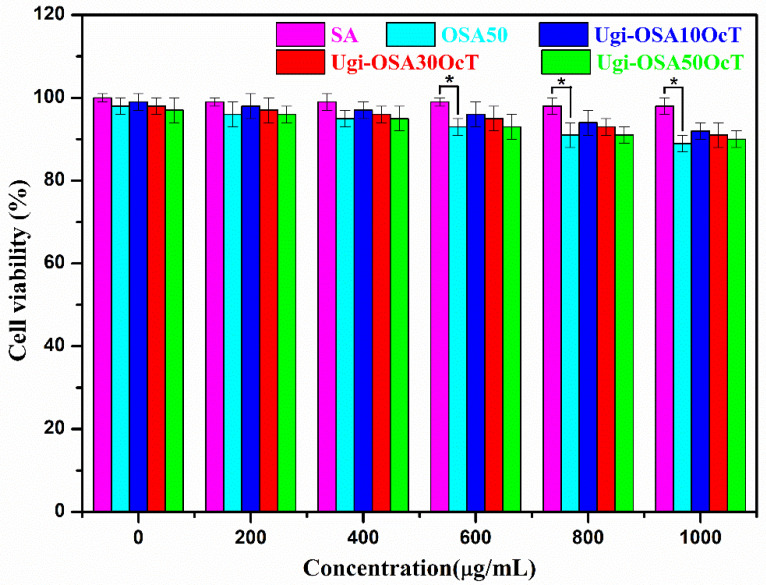
In vitro cytotoxicity of Ugi-OSAOcT conjugates, OSA and SA at various concentrations against RAW 264.7 cells after incubation for 48 h: * represents *p* < 0.05, indicating significant difference.

The in vitro cytotoxicity of the Ugi-OSAOcT conjugates, OSA and SA (control) against RAW 264.7 cells were evaluated by MTT assay. In general, a percentage of cell viability greater than 80% is considered to be a low cytotoxic in MTT assay analysis [73,74]. As shown in Figure 10, the Ugi-OSAOcT conjugates displayed a similar cell viability to SA; the cell viability of the RAW 264.7 cells remained above 90% after incubation for 48 h with a concentration of Ugi-OSAOcT conjugates ranging from 0 μg/mL to 1000 μg/mL. This indicated that an introduction of alkyl chains into OSA would not cause significant cytotoxicity against RAW 264.7 cells, even though the Ugi-OSAOcT concentration reached 1000 μg/mL. More importantly, the biocompatibility was independent of the concentration and DS of the Ugi-OSAOcT conjugates. These results were in good agreement with other studies on the development of amphiphilic OSA derivatives [75,76].

## 4. Conclusions

In the present study, amphiphilic Ugi-OSAOcT conjugates with different DS were successfully synthesized in distilled water at room temperature using OCA, OSA, HAc and TOSMIC via the Ugi-4CR for sustained release of IBU. The optimum reaction parameters were a reaction time of 12 h, a reaction temperature of 25 °C and a molar ratio of 1:2.4:3:3.3 (OSA:OCA:HAc:TOSMIC), respectively. The Ugi-OSAOcT conjugates could self-assemble into stable micelles in aqueous solutions with low CMC values in the range of 0.40–0.085 mg/mL, a small size in the range of 135.7 ± 2.4–196.5 ± 3.8 nm and negative zeta potential in the range of −32.8 ± 1.4–−38.2 ± 1.8 mV. The DL and EE of the IBU-loaded Ugi-OSAOcT micelles (IBU/Ugi-OSAOcT = 3:10, *w*/*w*) reached as much as 10.9 ± 0.4–14.6 ± 0.3% and 40.8 ± 1.6–57.2 ± 1.3%, respectively. The in vitro release study demonstrated that the IBU-loaded micelles exhibited sustained and pH-responsive drug release behavior, indicating that the micelles are suitable for oral drug delivery. In addition, the DS of the hydrophobic segment on the OSA backbone had an important effect on the IBU-loading and drug release behavior. Additionally, the Ugi-OSAOcT conjugates exhibited a lower cytotoxicity against RAW 264.7 cells, which indicates that the Ugi-OSAOcT micelles could be considered a safe carrier for biomedical applications. Therefore, it could be an ideal candidate for hydrophobic drug delivery in the biomedical field.

## Figures and Tables

**Table 1:** Elemental contents of Ugi-OSAOcT conjugates prepared by the optimum reaction parameters.

Sample ^a^	^b^ DO (%)	C (% m/m)	H (% m/m)	N (% m/m)	DS (%)
Ugi-OSA_10_OcT	9.51	51.5	7.45	0.89	4.8
Ugi-OSA_30_OcT	27.76	41.69	7.17	1.96	14.8
Ugi-OSA_50_OcT	44.25	30.36	6.77	2.1	24.3

^a^ Subscript refers to the degree of theoretical oxidation of OSA; ^b^ the degree of actual oxidation of OSA was determined by the hydroxylamine hydrochloride/sodium hydroxide colorimetric titration method.

**Table 2:** Optimization of reaction parameters for preparation of OCA-grafted OSA_10_ (Ugi-OSA_10_OcT) via Ugi-4CR.

N_OSA10_:N_OCA_:N_HAc_:N_TOSMIC_^a^	t (h)	T (°C)	DS (%) ^b^	Mw ^c^	Mn ^c^	Mw/Mn ^c^	Yield (%)
1:2:2:2.2	12	25	3.3	112,671	84,132	1.34	41.3
1:2.4:2.4:2.6	12	25	4.0	121,653	92,161	1.32	52.6
1:2.8:2.8:3.1	12	25	3.9	119,868	77,334	1.55	53.4
1:2.4:2.4:2.6	16	25	3.8	120,848	81,654	1.48	53.3
1:2.4:2.4:2.6	8	25	2.8	111,269	73,203	1.52	46.8
1:2.4:2.4:2.6	12	37	3.8	121,058	85,857	1.41	48.5
1:2.4:3:3.3	12	25	4.8	123,259	88,676	1.39	54.2
1:2.4:3.5:3.9	12	25	4.8	124,512	87,685	1.42	54.7

^a^ The molar ratio of reagent corresponding to OSA_10_:OCA:HAc:TOSMIC; ^b^ determined by EA; ^c^ determined by GPC (0.05% sodium azide as the mobile phase).

**Table 3:** Characteristics of Ugi-OSAOcT micelles (1.0 mg/mL): Data are presented as mean ± SD (*n* = 3).

Sample	DS (%)	CMC (mg/mL)	Size (nm)	PDI	Zeta Potential (mV)
Ugi-OSA_10_OcT	4.8	0.30	196.5 ± 3.8	0.43 ± 0.04	−38.2 ± 0.8
Ugi-OSA_30_OcT	14.8	0.20	178.3 ± 4.5	0.45 ± 0.03	−36.8 ± 0.6
Ugi-OSA_50_OcT	24.3	0.085	135.7 ± 2.4	0.37 ± 0.02	−32.8 ± 0.4

**Table 4:** Characteristics of IBU loaded Ugi-OSAOcT micelles. Data are presented as mean ± SD (*n* = 3).

Sample	Drug/Polymer (*w*/*w*)	DL (%)	EE (%)	Size (nm)	PDI	Zeta Potential (mV)
Ugi-OSA_50_OcT	1:10	3.9 ± 0.4	40.8 ± 1.6	142.5 ± 3.5	0.37 ± 0.06	−34.8 ± 1.4
Ugi-OSA_50_OcT	2:10	8.2 ± 0.5	44.6 ± 1.8	150.7 ± 2.3	0.41 ± 0.03	−35.2 ± 1.8
Ugi-OSA_50_OcT	3:10	14.6 ± 0.3	57.2 ± 1.3	160.3 ± 5.7	0.35 ± 0.02	−38.8 ± 0.6
Ugi-OSA_50_OcT	5:10	19.3 ± 1.2	52.4 ± 1.5	154.6 ± 4.8	0.36 ± 0.05	−36.7 ± 1.5
Ugi-OSA_10_OcT	3:10	10.9 ± 0.4	40.8 ± 1.6	210.8 ± 5.2	0.45 ± 0.03	−42.5 ± 0.3
Ugi-OSA_30_OcT	3:10	13.2 ± 0.5	50.6 ± 1.8	198.6 ± 4.8	0.43 ± 0.03	−42.3 ± 0.5

## Data Availability

The data presented in this study are available on request from the corresponding author. Samples of the compounds are available from the authors.
